# Comparative Genomics of *Escherichia coli* Serogroups 64474, O179, O188 and *Shigella boydii* O16

**DOI:** 10.3390/pathogens15050462

**Published:** 2026-04-24

**Authors:** Edwin Omar Desales-Decaro, Graciela Castro-Escarpulli, Andres Saldaña-Padilla, Alejandro Cravioto, Hugo G. Castelán-Sánchez, Armando Navarro-Ocaña

**Affiliations:** 1 Departamento de Microbiología, Escuela Nacional de Ciencias Biológicas, Instituto Politécnico Nacional, Mexico City 11340, Mexico; 2 Facultad de Medicina, Universidad Nacional Autónoma de México, Mexico City 04510, Mexico; dracravioto@hotmail.com; 3 Department of Pathology and Laboratory Medicine, Western University, London, ON N6A 5C1, Canada; 4 Public Health Department, Faculty of Medicine, Universidad Nacional Autónoma de México (UNAM), Avenida Universidad 3000, Ciudad Universitaria, Mexico City 04510, Mexico

**Keywords:** comparative genomics, *Escherichia coli*, *Shigella* spp., serogroup O-antigen, phylogenomics, pangenome analysis, clonality, virulence genes

## Abstract

*Shigella* spp., and *Escherichia coli* exhibit notable genomic and phenotypic similarities, including serologically and genetically related somatic antigens. For example, the relationship among pathogenic strains *E. coli* 64474, O179, O188, and *S. boydii* O16 suggests a shared clonal origin. To evaluate their genomic proximity, a comparative genomics study was conducted using whole-genome sequencing. Comparative genomics involved *rfb* gene cluster regions and whole-genome comparisons. Phylogenomic inferences were performed using the virtual genome fingerprint (VGF) method with bootstrap support. The results revealed a high degree of genomic similarity and a close evolutionary relationship among *E. coli* strains, which also demonstrated genetic associations with clinically relevant pathotypes through the presence of virulence genes. Furthermore, serogroups 64474, O188, and *S. boydii* O16 exhibited close genetic relationships, suggesting that serotype 64474 could represent a novel serogroup, although its similarity to O188 indicates the influence of divergent factors. These findings support the hypothesis that these *E. coli* strains originated from a common clonal lineage, enhancing our understanding of serogroup diversity and the evolutionary dynamics within enteric pathogens.

## 1. Introduction

*Escherichia coli* is a facultative anaerobic bacterium that includes both commensal and pathogenic strains; these pathogens are capable of causing a wide variety of diseases in different animal species Liu, 2020 [1]. Eight pathotypes that cause human diseases have been described, six of which are intestinal pathogens: enteropathogenic *E. coli* (EPEC), enterohemorrhagic *E. coli* (EHEC), enterotoxigenic *E. coli* (ETEC), enteroaggregative *E. coli* (EAEC), enteroinvasive *E. coli* (EIEC) and diffusely adherent *E. coli* (DAEC). The other two cause extraintestinal infections and are therefore called ExPEC; uropathogenic *E. coli* (UPEC) and meningitis-associated *E. coli* (MNEC) (Kaper, 2004; Pokharel, 2023) [2,3]. *Shigella* was recognized in 1890 as *Bacillus dysenteriae,* a facultative anaerobe intracellular pathogen that causes bacillary dysentery or shigellosis (Lan, 2002; Stenhouse, 2023) [4,5]. *E. coli* and *Shigella* spp., are closely related species that were part of the same genus (then called *Bacillus*) until 1950, when *Shigella* was assigned to its own genus and classified into four species: *S. boydii*, *S. dysenteriae*, *S. flexneri*, and *S. sonnei*, also known as subgroups A, B, C, and D, respectively (Stenhouse, 2023; Pupo, 2000) [5,6]. Since this taxonomic separation, classification systems for each genus have been developed independently, with somatic (O) antigen serotyping established to identify *Shigella* species, as they lack surface (K) and flagellar (H) antigens. In contrast, *E. coli* classification is mainly genotypic, although serotyping is also used, based on distinctions between O, K and H antigens [1,7]. Most genes involved in O-antigen biosynthesis are grouped in the *rfb* cluster [8], which is typically located between the *galF* and *gnd* genes in both *E. coli* and *Shigella* [1].

Strain identification of *E. coli* and *Shigella* spp., can be performed using biochemical and molecular tools; however, differentiation depends on the level of genetic relatedness and the purpose of the study. A high degree of genotypic and phenotypic similarity may complicate differentiation. It is common to find *E. coli* and *Shigella* spp. strains with similar O-antigen structures, which may lead to a misdiagnosis, particularly in infections causing bacillary dysentery—commonly associated with both *Shigella* spp., and EIEC due to their genetic similarity [9]. This is evident in the strains studied here: *E. coli* 64474, O179, O188, and *S. boydii* type 16 (*S*. *boydii* O16), which share epitopes, particularly *E. coli* 64474, O188 and *S. boydii* O16. These strains express highly similar O-antigen phenotypes and share some *rfb* genes, such as *wzx* (O-antigen flippase) and *wzy* (O-antigen polymerase) [10,11].

It is worth noting that *E. coli* 64474 strains have undefined O-antigen and were isolated from fecal specimens of pediatric patients with diarrhea in different countries [10]. Meanwhile, *E. coli* O188 has been associated with the antibiotic resistance genes *mcr-9.1*, *bla_VIM-1_*, *bla_KPC-3_*, which confer resistance to colistin and beta-lactams, respectively. These findings are relevant given the high agglutination similarity of these serotypes with the O-antigen of *S. boydii* O16, which may also harbor or acquire these genes [12]. Lastly, *E. coli* O179 is a Shiga toxin-producing strain (STEC) [13], and although it shows lower agglutination levels, it remains relevant to this study.

The aim of this study is to explore the genomic relatedness and evolutionary relationships among these strains through comparative genomics and phylogenomic analysis, to assess the existence of clonality.

## 2. Materials and Methods

### 2.1. Strains

*E*. *coli* 64474, O179:H8 (E43478) and *S*. *boydii* O16 (G1219) strains were obtained from a previous study by the laboratory [10] and *E*. *coli* O188:H10 was obtained from the Statens Serum Institut (Copenhagen, Denmark).

### 2.2. Serotyping

The *E*. *coli* 64474, O179, *E*. *coli* O188 and *S*. *boydii* O16 strains were serotyped by agglutination assays [14] using 96-well microtiter plates with rabbit antisera (SERUNAM) obtained against 188 somatic antigens and 53 flagellar antigens for *E. coli*, and against 45 somatic antigens for *Shigella* species. Rabbit serum against the *E*. *coli* O188 strain was also prepared, and the anti-*E*. *coli* 64474, O179, and *S*. *boydii* O16 sera were previously obtained from the study referenced above.

### 2.3. Absorption Assays

Rabbit sera prepared against *E*. *coli* 64474, O179, O188 and *S*. *boydii* O16 strains were absorbed with homologous and heterologous antigens according to the method described by Ewing [15].

### 2.4. DNA Extraction and Sequencing

Genomic libraries were prepared using the Illumina TruSeq DNA Nano protocol, with an average insert size of approximately 550 bp. Library quality was assessed using a High Sensitivity Bioanalyzer chip. Sequencing was performed in a paired-end 2 × 300 bp format. The i7 index sequences assigned to the four libraries were ATCACG, CGATGT, TTAGGC, and TGACCA, respectively. In addition, A-tailing was performed prior to adapter ligation. The Index 1 (i7) adapter sequence was (A)GATCGGAAGAGCACACGTCTGAACTCCAGTCAC[i7]ATCTCGTATGCCGTCTTCTGCTTG, where the base in parentheses corresponds to the additional **A** incorporated during A-tailing. For adapter trimming, the following sequences were used: Read 1, AGATCGGAAGAGCACACGTCTGAACTCCAGTCA; Read 2, AGATCGGAAGAGCGTCGTGTAGGGAAAGAGTGT. Genomic sequencing was performed by the Genomic Services Laboratory of the Center for Research and Advanced Studies of the IPN (CINVESTAV) Irapuato, Mexico.

### 2.5. Genome Assembly

The four genomes of *E*. *coli* 64474, O179, O188, and *S*. *boydii* O16 strains were sequenced using paired-end reads on an Illumina HiSeq 1500 platform. Raw reads (R1/R2) were evaluated with FastQC v0.12.1 and summarized with MultiQC v1.17. Reads were trimmed using Trimmomatic v0.39 to remove adapters/primers (ILLUMINACLIP), trim low-quality bases at read ends (LEADING:30, TRAILING:30), apply sliding-window trimming (SLIDINGWINDOW:4:15), and discard reads shorter than 75 bp (MINLEN:75). Post-trimming quality was re-assessed with FastQC v0.12.1. Genome assembly was performed with SPAdes v3.15.5 [16] using the “careful” and “cov-cutoff” options. Annotation was carried out with Prokka v1.14.6 [17], specifying genus, species, kingdom, and the selected database.

### 2.6. Core Genome and Pan-Genome Analysis

Comparative genomic analysis was performed using Roary v3.13.0 to identify core and accessory genes across all genomes included in this study. A total of *n* complete genome assemblies representing *Escherichia*, *Shigella*, and *Salmonella enterica* lineages were retrieved from the NCBI RefSeq database (accession numbers listed in Supplementary Table S1) to provide phylogenetic context. These publicly available reference genomes were downloaded in FASTA format and combined with four newly sequenced isolates generated in this study.

Protein clustering was performed using BLASTp with a minimum sequence identity threshold of 95%. The core gene alignment generated by Roary v3.13.0 was used to reconstruct a maximum-likelihood phylogeny using IQ-TREE2, with the best-fit substitution model selected according to the Bayesian Information Criterion (BIC). Branch support was assessed using the SH-like approximate likelihood ratio test (SH-aLRT) and ultrafast bootstrap approximation (UFBoot) with 1000 replicates. *Salmonella enterica* subsp. *enterica* serovar Enteritidis strain LA5_775 was used as the outgroup for tree rooting.

### 2.7. Virulence Genes

Specific virulence genes were identified by manually inspecting the annotation files generated with Prokka [17]. This process was guided by previously reported associations in the literature. In particular, the study by Navarro-García et al. [18] was used as a reference for the presence and characterization of virulence factors, including genes related to the Type VI secretion system [19,20]. The genes of interest included *stx2a*, *stx2b*, *sigA*, *pic*, and *iha*.

## 3. Results

### 3.1. Serotyping and Antigenic Cross-Reactions

Serotyping of the *E. coli* strains with 188 anti-O *E*. *coli* and 45 anti-O *Shigella* sera, showed positive agglutination reactions with *E*. *coli* 64474, O179, O188 and *S*. *boydii* O16 rabbit antisera prepared against the homologous O antigens.

### 3.2. Absorption Assays

To evaluate the presence of common epitopes among *E. coli* 64474, O179, O188, and *S. boydii* O16 strains, serum samples prepared against *E. coli* 64474, O179, O188, and *S. boydii* O16, were absorbed with homologous and heterologous antigens. The agglutination reactions were determined using the OX188 antiserum against *E*. *coli* O179, 64474, OX188 and *S. boydii* O16 antigens, the registered titers were 1:100, 1:800, 1:1600 and 1:400 respectively (Table 1). Absorption of OX188 antiserum with *E*. *coli* O179 antigen removed the reaction against the O179 antigen only. In contrast, when OX188 antiserum was absorbed with either *S*. *boydii* O16 or *E*. *coli* 64474 antigens, agglutination was completely removed for *E*. *coli* O179, *S*. *boydii* 16 and *E*. *coli* 64474 antigens, which means that OX188 shares a somatic antigen with *E*. *coli* 64474 and *S*. *boydii* O16.

Regarding to the *E*. *coli* 64474 strain, we previously reported that it shares a common O antigen with *S. boydii* O16 [10]. In that study, we proposed that *E*. *coli* 64474 serotypes belong to a new pathogenic serogroup showing a somatic antigen identical to that of *S*. *boydii* O16. The absorption assays of anti-*E*. *coli* 64474, anti-*S*. *boydii* O16, and anti-*E*. *coli* OX188 sera with 64474 and *S*. *boydii* O16 antigens showed that the agglutination reactions were completely removed against these antigens (Table 1). The results suggest that the *E*. *coli* OX188 strain shares a common O antigen with *E*. *coli* 64474 and *S*. *boydii* O16, and shares an antigenic fraction with O179, since the absorption assay of the antiserum of *E*. *coli* OX188 with O179 antigen removed only the reaction against the O179 antigen.

### 3.3. Comparative Genomics Analysis of rfb

**Figure 1:**
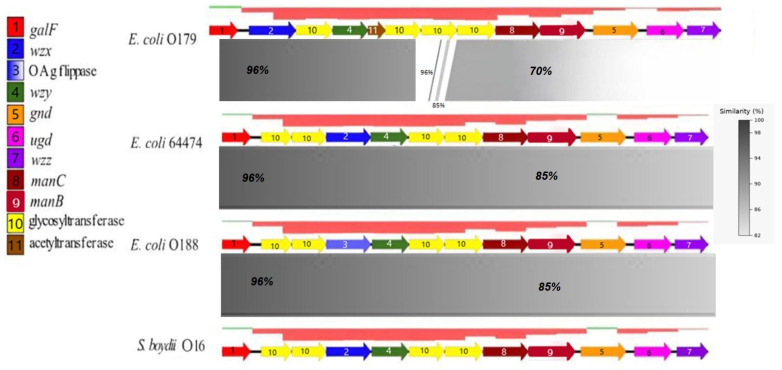
Comparative analysis of the rfb gene clusters in the four strains and their GC content. Each gene alignment is represented by a colored arrow as indicated in the legend on the left side. The percentage of similarity scale between aligned regions is shown in the lower right corner, while GC content is depicted above each gene cluster (green: positive values; red: negative values). Alignments were performed using BLAST v. 2.14.1 from Easyfig v. 2.2.2.

The results identified three distinct regions within the *rfb* cluster when comparing of *E. coli* O179 and *E. coli* 64474 (Figure 1). The first region, which contains the *galF* gene, showed similarity levels above 96%. The second region, located in the central part of the cluster, showed scattered short segments with similarity over 80%; however, these are not displayed in the figure due to their limited length. The third region extends from the glycosyltransferase gene upstream of *maC* to the *wzz* at the end of the cluster and shows a similarity greater than 70%. Three additional loci corresponding to *S. boydii* O16, *E. coli* 64474, and O188 shared similarity levels greater than 85%. In O188, the flippase gene matched the *wzx* gene, and it was arranged in the same order as in *S. boydii* O16 and *E. coli* 64474.

### 3.4. The Comparative Genomics Analysis

**Figure 2:**
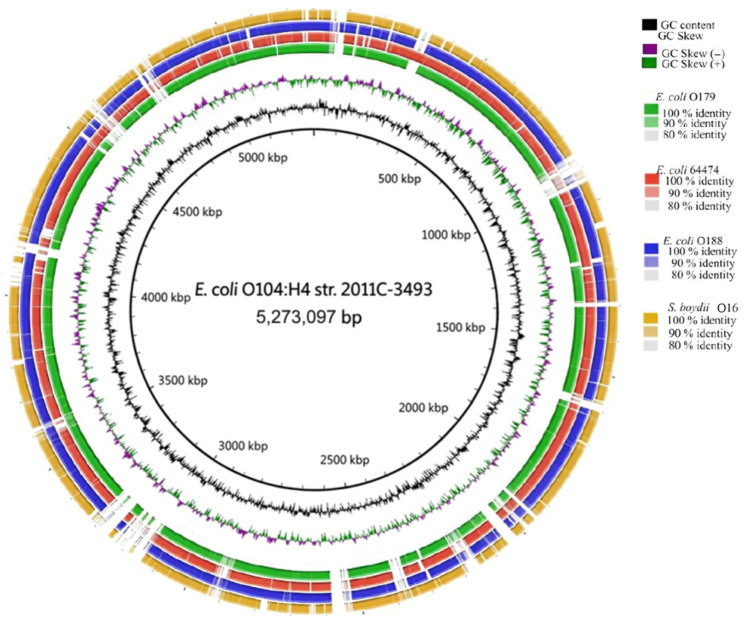
Comparative genomics of strains against a reference genome of *E. coli* O104:H4 strain 2011C-3493 (GCF_000299455.1). The analyses and their corresponding color codes are shown in the upper-right corner. The position along the reference genome is indicated in kilobase pairs (kbp) in the center pf the circular map. Alignments were performed using BLAST v. 2.14.1 via BRIG v. 0.95.

Comparison of the whole genome of *E. coli* O104:H4 strain 2011C-3493 with the study strains revealed regions with sequence similarity above 80%. Regions with 90% and 100% similarity are highlighted using distinct color labels. Additionally, GC content and GC skew are represented graphically (Figure 2).

### 3.5. Pangenome Structure and Core Genome Phylogeny

Analysis of the pangenome of *E. coli* 64474, O179, O188, and *S. boydii* O16 was performed using Roary v3.13.0 with a 95% BLASTp identity threshold. A total of 5199 gene clusters were identified across the four genomes. Of these, 3583 (68.9%) were classified as core genes, defined as those present in ≥99–100% of the genomes analyzed (i.e., all four strains), while the remaining 1616 (31.1%) constituted the accessory genome.

**Figure 3:**
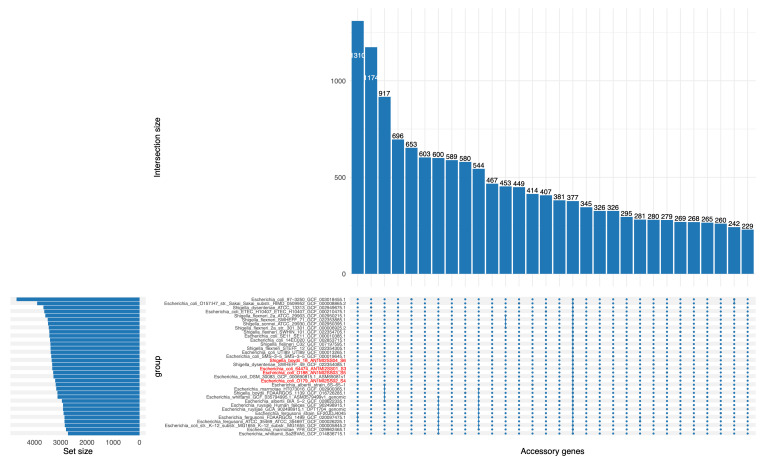
Roary-based pangenome structure and identification of genes present or absent in different genome sets. The UpSet plot shows the occurrence of gene clusters in *E. coli* 64474, O179, O188, and *S. boydii* O16 using Roary software version 3.13.0 with a BLASTp identity threshold of ≥95%. The top bar chart displays the number of gene clusters found in each combination of genome sets, while the left bar chart shows the total number of genes in each individual genome. The pangenome contains 5199 gene clusters, including 3583 core genes (gene clusters present in ≥99–100% of genome sets, i.e., all four genomes) and 1616 accessory genes. An UpSet plot highlighting a subset of gene clusters found in all four genomes (*n* = 377) is included as a representative portion of the core genome.

Figure 3 illustrates gene presence–absence patterns across the four genomes using an UpSet plot. Most accessory gene clusters are shared by only one or two genomes, indicating substantial genomic variability among strains. Notably, although 3583 gene clusters were classified as core genes, only a subset (*n* = 377) is represented as shared across all genomes in the UpSet plot. This discrepancy reflects the structure of the visualization, which does not display the full set of core genes but rather the subset captured in the plotted presence–absence matrix. Overall, the high proportion of core genes indicates the presence of a conserved genomic backbone among the four strains, supporting their close evolutionary relationship. In contrast, the accessory genome reflects lineage-specific variation, likely driven by horizontal gene transfer, prophage integration, and other mobile genetic elements contributing to genomic diversification.

The high proportion of shared core genes indicates a strong conserved genomic backbone among the four strains, supporting their close evolutionary relationship. In contrast, the accessory genome comprised genes variably distributed among strains, likely reflecting horizontal gene transfer events, prophage insertions, and other mobile genetic elements that contribute to genomic diversification.

To contextualize these findings within a broader evolutionary framework, a core genome alignment generated by Roary was used to reconstruct a maximum-likelihood phylogeny using IQ-TREE2. The tree was rooted with *Salmonella enterica* subsp. *enterica* serovar Enteritidis strain LA5_775.

**Figure 4:**
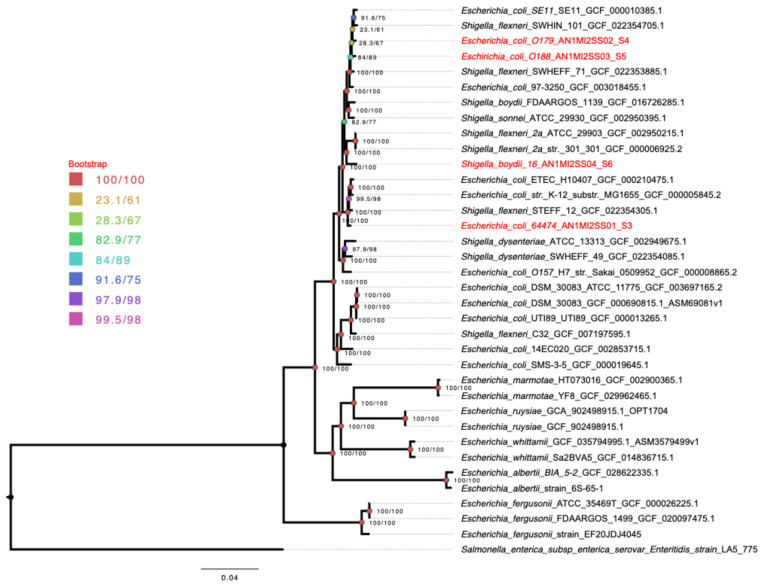
Core genome maximum-likelihood phylogeny inferred from Roary alignment. Phylogenetic tree reconstructed from the core gene alignment generated by Roary v3.13.0 and inferred using IQ-TREE2 under the best-fit substitution model selected according to the Bayesian Information Criterion (BIC). Branch support was assessed using SH-like approximate likelihood ratio test (SH-aLRT) and ultrafast bootstrap approximation (UFBoot) with 1000 replicates, and support values (SH-aLRT/UFBoot) are indicated at the nodes. The tree was rooted with *Salmonella enterica* subsp. *enterica* serovar Enteritidis strain LA5_775. Our sequenced strains are highlighted in red, while reference strains are shown in black. The topology illustrates the interspersed distribution of *E. coli* and *Shigella* lineages within a single major clade.

The phylogenetic tree reconstruction shows that *E*. *coli* and *Shigella* species are not differentiated into distinct, separate phylogenetic groups. Instead, they form a single, interspersed clade. This finding is consistent with the current genomic organization of these species and their relatedness. Within this clade, *E. coli* O179 and *E. coli* O188 are grouped together with *E. coli* SE11 and other related isolates. *E*. *coli* 64474 is also part of a single clade with other *E*. *coli* strains, indicating no differentiation from established *E*. *coli* lineages. In contrast, *S*. *boydii* O16 is grouped with *S*. *boydii* FDAARGOS_1139 and other related *Shigella* species, including *S*. *flexneri* and *S*. *sonnei* (Figure 4). In summary, all these isolates are part of a single clade with known species of the *Escherichia*/*Shigella* complex.

### 3.6. Virulence Genes

To better contextualize the biological relevance of the analyzed strains, we screened a panel of virulence genes representative of different *E. coli* pathotypes. The resulting profiles showed that the strains harbor markers commonly associated with STEC, UPEC, EAEC, ETEC, and *Shigella*-related virulence schemes, suggesting a notable overlap of pathogenic traits among them. In particular, some strains carried combinations of genes that are typically linked to more than one pathotype, supporting the idea that these isolates may display a hybrid virulence profiles (Table 2). At the strain level, *E. coli* O179 carried *stx2a*/*stx2b*, *iha*, and *sigA*, consistent with virulence traits associated with STEC and UPEC. In contrast, *E. coli* O188 harbored *sigA*, *pic*, *aggR*, *aatA*, and *aap*, a profile mainly related to EAEC and also shared with virulence schemes reported in STEC. Likewise, *E. coli* 64474 carried *eltA*/*eltB* and *aggR*, indicating a combination of ETEC- and EAEC-associated markers. Overall, these findings support the presence of diverse and partially overlapping virulence repertoires among the analyzed strains.

## 4. Discussion

In a previous study conducted in our laboratory, we showed that *E*. *coli* 64474 and *S*. *boydii* O16 strains exhibited similar agglutination reactions with the anti-O sera of the these strains. They also share the *wzx* (flippase) and *wzy* (polymerase) genes, which are involved in biosynthesis of the O antigen of *S*. *boydii* O16 [10,25]. These results suggested that these strains could belong to the same clone. In the present study, we found that the anti-O sera of *E*. *coli* 64474 and anti-*S*. *boydii* O16 reacted with the O antigen of *E*. *coli* O188. To elucidate whether these strains belonged to the same clone due to their similarities at the O antigen level, we performed a comparative genomics and phylogenomic analyses among *E*. *coli* 64474, *E*. *coli* O179, *E*. *coli* O188 and *S*. *boydii* O16.

There are various types of analyses used to distinguish strains of enterobacteria; however, phylogenomic analyses can differentiate closely related strains because they use a larger amount of genetic information. The phylogenomic analysis revealed a monophyletic clade formed by *E. coli* strains, indicating a close clonal relationship. *E. coli* O188 and O179 were the most closely related, while *E. coli* 64474 was positioned third, contrary to previous serological studies, which showed a closer relationship between serotypes O188 and 64474 and between serotypes O179 and 64474. These discrepancies were supported by the comparative analysis of the rfb clusters, where distinct genes were only found in a section of the O179 O-antigen cluster.

### 4.1. rfb Cluster

Discrepancies in the high-similarity patters between the O-antigen and whole-genome sequences of the strains may result from the high rate of homologous and non-homologous recombination within O-antigen cluster [26]. These recombination events can lead to changes in the entire genes or gene sets within these genomic hotspots [27], which are associated with integration sites [28]. When comparing the *rfb* cluster of 64474 and O179 strains, more differences were observed than when comparing 64474 with O188 and *S*. *boydii* O16. Notably, mutations were found in housekeeping genes such as *galF* [29], over 96% similarity, *gnd* [30], and *ugd* [31], these last two genes with over 70% of similarity, which are common within these clusters due to frequent genetic rearrangement. The O179 *rfb* cluster consists of a different gene set compared to the other three *rfb* clusters, explaining the lower serological similarity. Despite these differences, certain loci within the O179 cluster retained high levels of similarity (over 70%), which may indicate a recombination event between O179 and other genomically similar strains [32].

Although *S. boydii* O16 did not exhibit a close clonal relationship with the *E. coli* strains, comparison of their O-antigen clusters revealed high similarity (over 85%) among the *S. boydii* O16, O188 and 64474 clusters. This genomic similarity supports the idea of *S. boydii* O16 is evolutionarily related strain to *E. coli* strains of the study. Furthermore, in addition to O antigen and whole-genome similarities, other studies, such as the structural analysis of O188 have revealed only minor differences from *S. boydii* O16, specifically in the N-acetylated residues of LPS [11]. Some reports also suggest that genome similarities with *E. coli* O188 may indicate that *S. boydii* O16 could act as a gene receptor or donor, potentially acquiring genes that increase virulence or drug resistance [12].

The high similarity among rfb clusters of 64474, O188, and *S. boydii* O16 is consistent with the serological findings. Nevertheless, O antigen molecular structures cannot be inferred solely from genome sequences; therefore, these results must be supported by further analyses. Due to the complexity of these polysaccharides, structural characterization using bioinformatics tools combined with NMR spectroscopy may help to determine whether 64474 carries a de novo O antigen [33].

### 4.2. Comparative Genomics

Although this analysis revealed similarity below 80%; these regions were located at conserved positions across the genomes, showing shared patterns that support the high similarities among the genomes of the study strains. These loci include accessory genes related to phages, mobile elements, or sequences acquired through horizontal gene transfer (HTG), such as genes involved in the *mer* operon (a mercury resistance system) [34], the *sil* operon (which promotes resistance to exogenous silver) [35], and the Type VI Secretion System (or T6SS) [20]. However, most of the genomic elements belong to the core genome (68.9% of genes), as computed from *E*. *coli* 64474, O179, O188 and *S*. *boydii* O16 strains, indicating a common evolutionary pattern. When comparing this analysis with broader studies, a report analyzing over 95,525 genome sequences from 14 phylogroups of *E. coli* and *Shigella* spp., revealed a core genome of only 1.96%, highlighting the close evolutionary relationship of the strains studied [36,37].

### 4.3. Virulence Genes

In addition, virulence genes should be considered when assessing the significance of this relationship. Particularly informative are the similarity levels detected among *E. coli* 64474, *E*. *coli* O179, *E*. *coli* O188, *S. boydii* O16, and *E. coli* O104:H4 strain 2011C-3493, this last strain a clinically relevant strain responsible for the 2011 outbreak in Europe, which resulted in 46 deaths, 782 cases of hemolytic uremic syndrome, and 3128 cases of acute gastroenteritis. This strain is genetically related to the EAEC and STEC pathotypes and harbors a broad repertoire of virulence genes. In the analysis, virulence genes recognized as critical for this pathogen’s virulence *stx2a*, *stx2b*, *sigA*, *pic*, and *iha* [18] were found in *E. coli* O179 and O188. All these genes, whether present or absent, are associated with horizontal gene transfer (HGT), which may explain the presence of unrelated loci between the strains of the study and *E. coli* O104:H4 strain 2011C-3493 because those are not core genes [38]. This information is relevant because the genome similarity observed between *E. coli* O179 and O188 includes shared high-virulence genes with *E. coli* O104:H4 strain 2011C-3493. Moreover, *E. coli* 64474 and *S. boydii* O16 strains, may acquire these genes via genetic recombination, due to their close genetic relationship.

## 5. Conclusions

*E. coli* 64474 may possess a de novo O-antigen and be a clone more closely related to *E. coli* O188 and O179. However, additional factors must be considered, such as the isolation date (1985), geographic origin (Mexico), and genome content similarities that could have led to divergence events over time, ultimately affecting phylogenomic interpretations and potentially indicating that this strain descends from an ancestor with the *E. coli* O188 antigen.

Similarity levels of rfb clusters should be interpreted alongside other factors, such as genomic plasticity, HGT, homologous recombination [39], and epigenetic effects [40], as well as the presence of virulence and drug resistance genes that may be transferred through HGT mechanisms. These characteristics, frequently observed in *E. coli* and Shigella spp., enhance their adaptability and, in some cases, their pathogenicity [41,42].

## Figures and Tables

**Table 1:** Agglutination Titers of Absorbed and Unabsorbed *E. coli* O179, 64474:H32, *S. boydii* O16 and OX188 Sera.

	Titers of Unabsorbed Sera			Titers of Antisera Absorbed with Boiled Cultures						
Antigen	*E. coli* 64474:H32	*S. boydii* O16	*E. coli* OX188:H10	*E. coli* 64474:H32 Absorbed with		*S. boydii* O16 Absorbed with		*E. coli* OX188:H10 Absorbed with		
				*E. coli* O179	*S. boydii* O16	*E. coli* O179	*E. coli* 64474	*E. coli* O179	*S. boydii* O16	*E. coli* 64474
*E*. *coli* O179	1:800	1:800	1:100	-	-	-	-	-	-	-
*E. coli* 64474:H32	1:1600	1:3200	1:800	1:1600	-	1:1600	-	1:400	-	-
*E. coli* OX188:H10	1:800	1:1600	1:1600	1:800	-	1:800	-	1:800	-	-
*S*. *boydii* 16	1:3200	1:6400	1:400	1:800	-	1:3200	-	1:400	-	-

*E*. *coli* 64474, *E*. *coli* O179:H8 (E43478) and *S*. *boydii* O16 (G1219) strains were obtained from a previous study by the laboratory [10] and *E*. *coli* O188:H10 was obtained from the Statens Serum Institut (Copenhagen, Denmark).

**Table 2:** Virulence genes identified in the analyzed strains and their association with pathogenic *E*. *coli* and *Shigella* spp.

Gene	Strain	Association	Function
*stx2a stx2b*, *iha* *sigA*	*E. coli* O179	STEC, UPEC	Stx: Shiga toxin subunit A Stx2c and Shiga toxin subunit B Stx2a [19,21]. Iha adhesin: Bifunctional siderophore receptor [22]. SigA: serine protease autotransporter toxin [23].
*sigA* *pic* *aggR* *aatA* and *aap*	*E. coli* O188	EAEC, *Shigella* spp.	SigA: serine protease autotransporter toxin [23]. Pic: protein involved in intestinal colonization, serine protease autotransporter toxin [24]. *AggR*: transcriptional activator from EAEC of virulence factors. *aatA*: Facilitate the export of the dispersin Aap protein across the outer membrane. *aap* (dispersin): It functions in dispersion over the surface of the intestinal mucosa
*eltA*/*eltB, aggR*	*E. coli* 64474	ETEC, EAEC	Lt: Activates intracellular adenylate cyclase [3]. *AggR*: transcriptional activator from EAEC of virulence factors

EAEC: Enteroaggregative *E. coli*; STEC: Shiga toxin-producing *E. coli*; UPEC: Uropathogenic, *E. coli*; Enterotoxigenic *E. coli*: ETEC.

## Data Availability

The data supporting the findings of this study are available from the corresponding authors upon reasonable request.

## Supplementary Materials

The following supporting information can be downloaded at: https://www.mdpi.com/article/10.3390/pathogens15050462/s1, Table S1: Genomes analyzed in this study and their GenBank accession numbers.
