# Supplementary material for: Comparative Genomics of Escherichia coli Serogroups 64474, O179, O188 and Shigella boydii O16
Source: Pathogens. 2026 Apr 24;15(5):462. doi: 10.3390/pathogens15050462 (PMC13209904; doi:10.3390/pathogens15050462)
Supplement: Supplementary file 1 [file pathogens-15-00462-s001.zip › pathogens-4255417-supplementary.pdf]

Table S1. Genomes analyzed in this study and their GenBank accession numbers

Strain	Access number
***Shigella flexneri* SWHIN_101**	GCF_022354705.1
***Escherichia coli* SE11**	GCF_000010385.1
***Escherichia coli* O179_AN1M2SS02_S4**	This study
***Escherichia coli* O188_AN1M2SS03_S5**	This study
***Shigella flexneri* SWHEFF_71**	GCF_022353885.1
***Escherichia coli* 97–3250**	GCF_003018455.1
***Shigella sonnei* ATCC_29930**	GCF_002950395.1
***Shigella boydii* FDAARGOS_1139**	GCF_016726285.1
***Shigella flexneri* 2a_str_301**	GCF_000006925.2
***Shigella flexneri* 2a_ATCC_29903**	GCF_002950215.1
***Shigella boydii* 16_AN1M2SS04_S6**	This study
***Escherichia coli* str_K–12_substr_MG1655**	GCF_000005845.2
***Escherichia coli* ETEC_H10407**	GCF_000210475.1
***Shigella flexneri* STEFF_12**	GCF_022354305.1
***Escherichia coli* 64474_AN1M2SS01_S3**	This study
***Shigella dysenteriae* ATCC_13313**	GCF_002949675.1
***Escherichia coli* O157**	GCA_000008865.2
***Shigella dysenteriae* SWHEFF_49**	GCF_022354085.1
***Escherichia coli* DSM_30083**	GCF_000690815.1
***Escherichia coli* DSM_30083 = JCM_1649 = ATCC_11775**	GCF_003697165.2
***Escherichia coli* UTI89**	GCF_000013265.1
***Shigella flexneri* C32**	GCF_007197595.1
***Escherichia coli* 14EC020**	GCF_002853715.1
***Escherichia coli* SMS–3–5**	GCF_000019645.1
***Escherichia fergusonii* FDAARGOS_1499**	GCF_020097475.1
***Escherichia fergusonii* ATCC_35469**	GCF_000026225.1
***Escherichia fergusonii* strain EF20JDJ4045**	GCF_000191665.1
***Escherichia russiae* Human_faeces**	GCF_902498915.1
Escherichia russiae	GCA_902498915.1
***Escherichia marmotae* YF8**	GCF_029962465.1
***Escherichia marmotae* HT073016**	GCF_002900365.1
***Escherichia whittamii* Sa2BVA5**	GCF_014836715.1

Table S1. Genomes analyzed in this study and their GenBank accession numbers

Escherichia whittamii	GCF_035794995.1
Escherichia albertii **strain 6S-65-1**	GCF_035658295.1
Escherichia albertii **BIA_5–2**	GCF_028622335.1
Salmonella enterica **subsp.** ***enterica*** **serovar Enteritidis strain LA5_775**	GCF_000313715.1
